# The Role of TLRs in Anti-cancer Immunity and Tumor Rejection

**DOI:** 10.3389/fimmu.2019.02388

**Published:** 2019-10-22

**Authors:** Zuzanna Urban-Wojciuk, Mohd M. Khan, Benjamin L. Oyler, Robin Fåhraeus, Natalia Marek-Trzonkowska, Aleksandra Nita-Lazar, Ted R. Hupp, David R. Goodlett

**Affiliations:** ^1^International Centre for Cancer Vaccine Science (ICCVS), University of Gdaǹsk, Gdaǹsk, Poland; ^2^Laboratory of Immune System Biology (LISB), National Institute of Allergy and Infectious Diseases (NIAID), National Institutes of Health (NIH), Bethesda, MD, United States; ^3^University of Maryland School of Medicine, Baltimore, MD, United States; ^4^Department of Medical Biosciences, Umeå University, Umeå, Sweden; ^5^Université Paris 7, INSERM, UMR 1162, Paris, France; ^6^Regional Centre for Applied Molecular Oncology, Masaryk Memorial Cancer Institute, Brno, Czechia; ^7^Laboratory of Immunoregulation and Cellular Therapies, Department of Family Medicine, Medical University of Gdaǹsk, Gdaǹsk, Poland; ^8^Cell Signaling Unit, Institute of Genetics and Molecular Medicine, University of Edinburgh, Edinburgh, United Kingdom; ^9^Department of Microbial Pathogenesis, University of Maryland School of Dentistry, Baltimore, MD, United States

**Keywords:** toll-like receptors, immuno-oncology, anti-cancer immunity, tumor rejection, immunotherapy

## Abstract

In recent years, a lot of scientific interest has focused on cancer immunotherapy. Although chronic inflammation has been described as one of the hallmarks of cancer, acute inflammation can actually trigger the immune system to fight diseases, including cancer. Toll-like receptor (TLR) ligands have long been used as adjuvants for traditional vaccines and it seems they may also play a role enhancing efficiency of tumor immunotherapy. The aim of this perspective is to discuss the effects of TLR stimulation in cancer, expression of various TLRs in different types of tumors, and finally the role of TLRs in anti-cancer immunity and tumor rejection.

## Introduction

Toll-like receptors (TLRs) play a key role in the activation of innate immunity due to their ability to recognize highly conserved molecules expressed by pathogens. Alongside pathogen-associated molecular patterns (PAMPs), TLRs also recognize endogenous ligands (alarmins, also called danger-associated molecular patterns or DAMPs). Alarmins are excreted by cells upon tissue injury or cell death, but their excessive release is associated with autoimmune diseases and cancer (1, 2). Cell death and chronic inflammation are key features of tumorigenesis leading to increased production of alarmins in many types of cancer such as breast, colon, pancreatic cancer, melanoma and glioblastoma (3–5).

TLRs can be located either on the cell membrane (TLR1, 2, 4, 5, 6) or on the membrane of endosomes within the cell (TLR3, 7, 8, 9) depending on the type of ligand they recognize. Thus, TLRs located on the cell membrane bind lipids and proteins, whereas TLRs located on the endosomal membranes bind nucleic acids (6). TLRs transmit signals through adaptor proteins to the nucleus leading to regulation of the innate and adaptive immune response. Since Deidier observed that patients infected with syphilis developed very few malignant tumors almost 300 years ago, it has been known that activation of the immune system by bacterial infection can cause cancer remission (7–9). However, cancer treatment with bacteria is rather controversial due to the potential toxicity (including flu-like symptoms but also septic shock) and a chance of bacteria mutating into antibiotic-resistant strains (10). Therefore, TLR ligands (bacterial components, as opposed to whole bacteria) have been mostly used in cancer therapy (11). On the other hand, the role of TLR expression in cancer cells is not very clear and has been correlated with either good or bad outcomes. Understanding the role of TLR activation in anti-cancer immunity and tumor rejection could accelerate the advancements in the field of immunotherapy and improve patients' survival.

## Expression of TLRs in Different Types of Cancer

TLRs are expressed by antigen presenting cells (such as dendritic cells and macrophages) as well as by fibroblasts and epithelial cells with their main role being host protection from microbial infection (12). However, functional TLRs are also present on cancer cells and their expression is often correlated with disease prognosis, as summarized in Table 1.

**Table 1:** Impact of TLR expression on patients' outcome.

**Cancer**	**TLR**	**Observation**	**References**
Esophageal cancer	TLR3, 4, 7, 9	TLR3, 4, 7, and 9 are overexpressed in esophageal cancer, TLR9 expression correlates with advanced stage, poor differentiation and high proliferation	(13, 14)
Lung cancer	TLR4, 5, 7, 8, 9	Expression of TLR4, 5, 7, 8, and 9 is higher in lung cancer than in normal cancer tissue; TLR5 is associated with good prognosis, TLR7 with poor clinical outcome	Reviewed in Gu et al. (15)
Melanoma	TLR7 and 8	High TLR7 and 8 expression is associated with high expression of immune cell markers and predicts longer overall survival	(16)
Pancreatic cancer	TLR7 and 8	TLR7 and 8 are highly expressed and stage-dependent in pancreatic cancer compared to normal pancreas	(17)
Breast cancer	TLR9	Low tumor TLR9 expression predicts shorter disease-free survival in triple-negative breast cancer (TNBC) patients	(18), reviewed in Sandholm and Selander (19)
Renal cell carcinoma	TLR9	TLR9 expression is associated with better survival	(20)
Glioma	TLR9	TLR9 expression increases according to the glioma grade, therefore high expression is associated with poorer survival TLR9 expression is elevated in glioblastoma stem cells	(21) (22)
Prostate cancer	TLR9	TLR9 expression is associated with decreased progression-free survival	(23)
Meta-analysis of 15 studies of solid tumors	TLR4	Elevated expression of TLR4 is associated with poor overall survival and shorter disease-free survival	(24)

Data presented in Table 1 underscores the versatility of TLR expression correlation with cancer prognosis. The same receptor can be associated with either good or bad prognosis (like TLR9) or can be generally correlated with a bad outcome (like TLR4). This makes TLRs difficult to study as a whole in the context of oncogenesis and cancer progression and suggests that researching single receptors in particular types of cancer may be a better approach. Different populations of cells (i.e., cancer stem cells, cancer cells, tumor-infiltrating lymphocytes, tumor-associated fibroblasts etc.) may have different TLR expression and, as a result, respond differently to TLR stimulation (25, 26).

One recent review discusses expression of TLRs in normal, pre-malignant and malignant epithelium of esophagus and oral cavity (27). It draws attention to TLR2, 4, and 5, which are normally expressed on cell membrane, but upon transformation toward dysplasia and cancer their expression increases and becomes more cytoplasmic. It is concluded that changes in TLR locations and their constitutive activation can lead to chronic inflammation and tumor progression, as opposed to transient inflammation leading to tumor eradication.

Recently, expression of TLRs, and direct pro-and anti-tumor effects of TLR ligands on cancer cells have also been studied (25). It was shown that glioblastoma stem cells have very low TLR4 expression in comparison to non-stem cells and don't respond to TLR4 stimulation, which allows them to survive despite immune signaling (25). The authors show direct link between TLR4 signaling and stemness, and suggest a treatment strategy based on TLR4 re-expression. Despite low TLR4 expression glioblastoma stem cells express high levels of TLR2, and its stimulation by high-mobility group box 1 (HMGB1) enhanced the stemness markers of those cells (28).

## Anti-Tumor Role of TLR Stimulation

Bacteria and their products have long been known to have anti-tumor properties (29). The initial work of Deidier was followed by Coley's development of sarcoma treatment with a mixture of bacterial toxins (30). Coley's results were not widely accepted by medical society due to inconsistencies and were not followed for a long time, these days, however, he's often called “Father of immunology” (8). Many years later, an outer membrane component of Gram-negative bacteria, lipopolysaccharide (LPS), was identified as an active fraction of the Coley's toxin, suggesting the involvement of TLR4 activation [reviewed in (7, 31)]. Due to systemic toxicity, LPS and other bacterial products must be administered locally, often in a form of an intra-tumoral injection. It was recently shown that an attenuated strain of *Clostridium novyi* efficiently decreased tumor size not only in a rat model but also in dogs with spontaneous solid tumors and one sarcoma patient (32). Such treatment is well-targeted as spores of *Clostridium novyi* germinate only within hypoxic regions of cancerous tissue and induce immune response probably via TLR activation (33). An attenuated strain of *Mycobacterium bovis* called Bacillus Calmette-Guérin (BCG), developed as a tuberculosis vaccine, has been used as a treatment for bladder cancer for over 40 years (34). The exact mode of action of BCG is unknown but its anti-cancer effect is caused by both direct effect of BCG infection on cancer cells as well as of immune response to it (35).

BCG, a TLR2/4 ligand, is one of the three FDA-approved TLR ligands. The others are the TLR4 ligand monophosphoryl lipid A (MPLA) and the TLR7 agonist imiquimod. Several TLR ligands have been shown to have an anti-tumor effect in different types of cancer; some key ones are listed in Table 2.

**Table 2:** TLR ligands as efficient anti-tumor agents in different models of cancer.

**TLR**	**Ligand**	**Cancer and model**	**Observation**	**References**
TLR2/4	Synthetic derivative of lipid A, OM-174	Melanoma (*in vivo*, syngeneic)	Reduces tumor progression, prolongs survival especially in combination with cyclophosphamide	(36)
	BCG	Bladder cancer (FDA-approved)	Reduces recurrence and prolongs survival of bladder cancer patients	(34), reviewed in Fuge et al. (35)
	BCG; OM-174; synthetic lipid A analog, ONO-4007; macrophage-activating lipopeptide (MALP)-2	Several syngeneic animal models and phase I clinical trial	Ligands alone or with chemotherapeutics induce Tumor necrosis factor alpha (TNFα) secretion, apoptosis and dendritic cell (DC) traffic	Reviewed in Garay et al. (7)
TLR3	Synthetic DNA/RNA hybrid molecule, ARNAX	Several mouse models, syngeneic and genetically engineered mouse models (GEMM) (*in vivo*)	TLR3 ligand as an adjuvant overcomes programmed death-ligand 1 (PD-L1) resistance without systemic cytokine/interferon production	(37)
TLR3 and TLR7/8	Hiltonol and resiquimod	Several (phase 1 clinical trial)	TLR ligands as adjuvants of DC vaccine (New York esophageal squamous cell carcinoma 1 (NY-ESO-1) tumor associated antigen targeted to DCs), both ligands are efficient	(38)
TLR4	MPLA	human papillomavirus (HPV)-induced cervical cancer (FDA-approved)	Potent vaccine adjuvants, promote type 1 T helper (Th1)-biased immune response	Reviewed in Gregg et al. (39, 40)
TLR5	Entolimod	Experimental and spontaneous liver metastases (*in vivo*, syngeneic)	Entolimod suppresses liver metastases and stimulates long-term antitumor T-cell immunity	(41)
TLR7	Imiquimod	Various cutaneous malignancies (FDA-approved for basal cell carcinoma)	Induction of apoptosis and stimulation of cell-mediated immune response	Reviewed in Bubna (42)
TLR7/8 and 9	Small molecule, 3M-052; and CpG oligodeoxynucleotides (CpG ODN)	Syngeneic colon cancer and melanoma cell lines (*in vivo*)	Combination of both agents eradicated large tumors and established long-term immunity by increasing number and activity of cytotoxic T cytotoxic T lymphocytes (CTLs) and natural killer cells (NK) cells	(43)
TLR7 and TLR9	Small molecule, 1V270; and CpG-C class ODN, SD-101	Head and neck squamous cell carcinoma (*in vivo*, syngeneic)	Intratumoral injection of TLR agonists activates tumor-associated macrophages (TAMs) and enhances the tumor suppressive effect of PD-L1 inhibition	(44)
TLR9	CpG ODN	Several types of cancer (Phase 1/2 clinical trials)	CpG ODNs enhance efficacy of immune checkpoint inhibitors	(45)

*In vivo and clinical trials denote experiments done in animal models and human subjects, respectively*.

Although TLR ligands can be efficient as a monotherapy, they are usually administered in a combined treatment, often playing the role of vaccine adjuvants (46). Their efficiencies as immunotherapeutic agents rely mostly on the initiation of T-cell immunity—antigen uptake, processing and presentation, maturation of dendritic cells, and activation of T cells (11). Briefly, PAMP/DAMP binding to TLR on immature antigen-presenting cells (APCs) induces their maturation to professional APCs that can present antigens (i.e., bacterial or cancer) on major histocompatibility complex I (MHC I). Antigens are presented to T-cells via T-cell receptor (TCR)-MHC I binding in the presence of co-stimulatory molecules such as cluster of differentiation 80 (CD80), CD86 (on antigen-presenting cells (APCs) binding CD28 (on T cells). Upon TCR activation, T cells produce CD154 that binds CD40 on APC surface leading to further activation of cells (both APCs and T cells). Lineage commitment of T cells depends also on the presence of transcription factor and cytokines; cytokines produced by T helper cells are often stimulating cytotoxic T cell activation (47, 48).

Beside expression on professional APCs TLRs can also be expressed in T cells where they act as co-stimulatory receptors that complement TCR-induced signaling to enhance T cell proliferation and cytokine production (49, 50). Additionally TLR stimulation of T regulatory cells may revert their immunosuppressive capabilities, which was shown for a synthetic TLR2 ligand (51, 52). This is particularly interesting for cancer research due to high levels of Tregs and their activity in tumor microenvironment.

TLR ligands as monotherapies have varied efficiency; there are several reports showing a modest effect of TLR stimulation in clinical trials, such as the TLR9 ligand CpG-ODN in glioblastoma (53) and the TLR7 ligand 852A in hematological malignancies (54). It remains to be defined why in those studies some patients responded very well to TLR ligands while others did not but may be caused by differences in TLR expression or immune infiltration within the tumor. A list of active clinical trials with TLR agonists used as adjuvants has been recently published (48).

## Pro-Tumor Role of TLR Ligands

As opposed to acute inflammation, chronic inflammation has been associated with tumor progression and is one of the hallmarks of cancer. Inflammatory cells secrete growth and survival factors, proangiogenic factors, extracellular-matrix modifying enzymes, and reactive oxygen species (ROS) that can lead to enhanced mutagenesis, growth, and invasion of cancer (55–57). Indeed, TLR4 stimulation by LPS increased production of immunosuppressive cytokines, possibly contributing to tumor immune escape, and induced resistance to apoptosis in lung cancer cells (58). It has also been shown that the LPS from *Helicobacter pylori* activates TLR4 on gastric cancer cell lines leading to increased proliferation (59). A study by Huang and colleagues underlines that one bacterial species can both increase and decrease tumor growth, depending on the route of administration (60). While intravenous *Listeria monocytogenes* vaccination inhibited tumor growth in mice, injection of bacteria directly into tumor mass promoted tumor growth possibly due to TLR2 activation on malignant cells.

TLR2 and TLR4 inhibition was shown to be efficient treatment for myeloid malignancies. Patients with myelodysplastic syndrome (MDS), a hematopoietic stem cell disorder that may lead to cancer, overexpress TLR2 and may benefit from TLR2 inhibition by OPN-305 antibody (61). It was also reported that MDS patients overexpress HMGB1 and its inhibition with sivelestat induces MDS cell death while spares healthy hematopoietic cells (62). CX-01, a synthetic TLR2/4 inhibitor, is currently in clinical trial for acute myeloid leukemia (63). Innate immune signaling, specifically TLRs expression in myelodysplastic syndromes is covered in detail elsewhere (64).

R848-stimulation of TLR7/8 overexpressing pancreatic cancer cell line resulted in increased cell proliferation and reduced chemosensitivity (17). The authors also show increased nuclear factor kappa-light-chain-enhancer of activated B cells (NFκB) and cyclooxygenase-2 (COX-2) expression upon TLR7/8 stimulation that has been previously linked to immune evasion and immunotherapy resistance (65).

**Figure 1:**
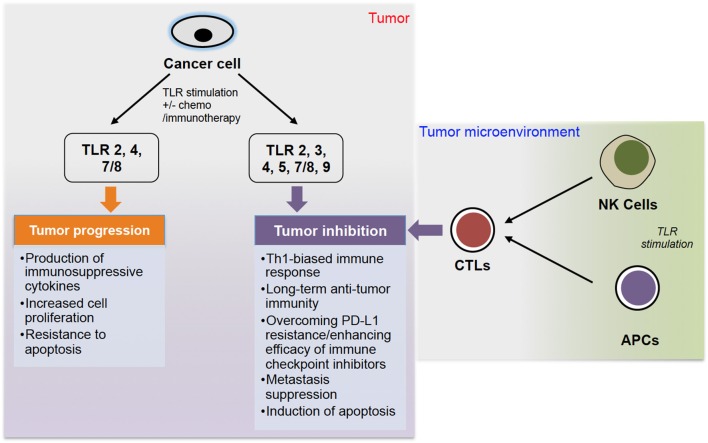
The role of TLR stimulation in cancer progression. TLR stimulation of cancer cells can lead to either tumor progression or inhibition. Stimulation of TLR 2, 4, and 7/8 can lead to tumor progression via production of immunosuppressive cytokines, increased cell proliferation and resistance to apoptosis. On the other hand, stimulation of TLR 2, 3, 4, 5, 7/8, and 9, often combined with chemo- or immunotherapy, can lead to tumor inhibition via different pathways. Additionally, stimulation of TLRs on NK cells and APCs (DCs and macrophages) can induce CTLs to further inhibit tumor growth.

Therefore, it seems that TLR signaling can act as a double-edged sword in cancer (summarized in Figure 1), with its pro- and anti-cancer roles that have been also reviewed by others (6, 66–68). A recent review by Braunstein et al. summarizes the clinical applications of TLR ligands, including recent clinical trials, but also gives a thorough introduction to TLR biology (69).

## Role of TLR Adaptor Proteins in Cancer

TLRs are bound to cell membranes, therefore TLR signaling is transduced via adaptor proteins such as myeloid differentiation primary response-88 (MyD88) and TIR-domain-containing adapter-inducing interferon-β (TRIF). MyD88 and TRIF signaling lead to expression of cytokines such as TNF-α, interleukin-1 beta (IL-1β), interleukin-6 (IL-6), interferon gamma-induced protein 10 (IP-10), and IFN-γ through the activation of transcriptional factors NF-κB, activator protein 1 (AP-1), and interferon regulatory factor 3 (IRF-3) (70). Additionally, MyD88 activation can signal via c-Jun N-terminal kinase (JNK) or extracellular signal-regulated kinase (ERK) signaling cascades leading to cell survival and proliferation. MyD88 can also signal independently of TLRs, through interleukin (IL)-1 receptor families (71). All TLRs except for TLR3 are signaling through MyD88 while TLR3 and some of TLR4 signaling is transmitted by TRIF (72). Although MyD88 can associate directly with TLRs, an additional protein called TIR-domain containing adaptor protein (TIRAP) has been shown to facilitate MyD88 interaction with TLR2 and TLR4 (73). TLR4 requires the presence of another adaptor, TRIF-related adaptor molecule (TRAM), to associate with TRIF (74).

Mice lacking MyD88 were developed in 1998 and have since been used to show the crucial role of MyD88 in resistance to bacterial or parasite infection (75–77). Signaling from TLRs and MyD88 is involved in protective inflammation responses to control gut bacterial numbers and intestinal epithelial cell homeostasis (71). However, the role of MyD88 in colon cancer development is more complicated. MyD88 was found to be a target for synthetic lethality in colon cancer; it acts as a bridge between inflammatory signaling pathways from TLRs and Ras oncogenic signaling (78, 79). MyD88 inhibition increased colon cancer cell line sensitivity to genotoxic agents *in vitro* and *in vivo*, reducing tumor growth and increasing apoptosis.

The most malignant subtype of diffuse large B-cell lymphoma (DLBCL) is associated with gain-of-function mutation in MyD88 (L265P) (80). This mutant form of MyD88 promotes cell survival by increasing NF-κB signaling and signal transducer and activator of transcription 3 (STAT3) activation. Because the mutation lies within the TIR domain of MyD88, it is suspected to either promote self-assembly or enhance the affinity of MyD88 to TLR TIR domains. The same activating MyD88 mutation also occurs in almost 3% of chronic lymphocytic leukemia cases (81). Based on the fact that Burton tyrosine kinase (BTK), a critical node in B-cell receptor signaling cascades, preferentially binds mutated MyD88, the BTK inhibitor ibrutinib is being tested in clinical trials for patients with several types of lymphoid malignancies (82). Furthermore, MyD88 L265P is a tumor-specific mutation, therefore it can elicit T-cell response which can be potentially used in immunotherapy.

Recently, MyD88 was shown to mediate sensitivity to histone deacetylase (HDAC) inhibition (83). High expression of MyD88 was associated with increased sensitivity to HDAC inhibitor SAHA resulting in decreased proliferation and enhanced cell death. Moreover, the authors show that HDAC inhibition is also efficient in DLBCL cell lines with L265P MyD88 mutation. This interesting link between epigenetics and innate immunology provides a plausible explanation for sensitivity of some malignancies to HDAC inhibition.

In high-grade serous ovarian carcinoma, elevated expression of MyD88 was associated with advanced stage and shortened overall survival (84). In the same cohort of patients strong TLR4 and MyD88 expression correlated with favorable survival in patients with low-grade serous ovarian cancer. Even though MyD88 inhibition was an efficient treatment in some cases of colon cancer (as previously mentioned), another group has shown that the mRNA level of *MyD88* is lower in cancerous colon tissue than in adjacent normal tissue (85). These two examples suggest that MyD88 may play different roles in various types and subtypes of cancer.

## TLRs in Anti-Cancer Immunity and Immune Rejection

Our immune system is trained very well to fight microbes such as bacteria and viruses. However, tumor cells are often too similar to normal cells to induce anti-cancer immunity and they have mastered the mechanisms of immune evasion. Triggering anti-cancer immunity should lead to efficient eradication of tumor cells and is a goal of many anti-cancer therapies.

The stroma of all solid tumors contains macrophages, which may either suppress or promote tumor development depending on their activation phenotype (86, 87). Classically activated macrophages (M1-activated macrophages) produce pro-inflammatory and immunostimulatory cytokines; they are crucial for host defense and tumor cell killing. The alternatively activated macrophages (M2) produce anti-inflammatory cytokines, promote angiogenesis and matrix remodeling leading to tumor progression and metasiasts. Several TLR agonists were shown to reprogram M2 macrophages into M1 type, which may be important in cancer immunotherapy (88). It was recently reported that TLR agonists synergize with interferons (type I or II) to induce antitumor M1 macrophages (89, 90). Another group combined TLR7/8 ligand R848 with anti-PD-1 therapy *in vivo* to see inhibition of tumor growth even in PD-1 resistant mice (91). Those studies point to another potential application of TLR ligands in immunotherapy and suggest that combination of TLR agonists with other immunomodulators could increase their efficiency when targeting macrophages.

The TLR5 ligand entolimod was shown to increase survival of mice with colorectal cancer metastases to the liver (41). This effect was caused by NK-cell-dependent activation of dendritic cells that led to stimulation of CD8+ T-cells. Interestingly, entolimod also induced formation of durable CD8+ T-cell memory sufficient for protection against tumor re-challenge. Although primarily efficient against liver metastases due to the restricted pattern of TLR5 expression and C-X-C motif chemokine receptor 3 (CXCR3)-dependent homing of NK-cells to the liver, the same mechanism led to entolimod suppression of lung metastases.

Many tumors constitutively express PD-L1 and therefore evade immune system surveillance. Targeting PD1/PD-L1 is a promising treatment strategy especially in immunogenic types of cancer such as melanoma and non-small cell lung cancer (NSCLC). However, many tumors remain unresponsive to it, mostly due to the lack of tumor-specific CD8+ T-cell infiltration (92, 93). The TLR3 agonist ARNAX was recently shown to be a potent PD-L1 blockade adjuvant, capable of overcoming resistance to PD-L1 inhibition *in vivo* (37, 94). ARNAX administered with tumor-associated antigen (TAA) triggers maturation of dendritic cells, which then present TAAs, therefore inducing anti-tumor CTLs without systemic cytokine/interferon production, leading to tumor regression. Interestingly, even without addition of TAA, ARNAX decreased tumor growth, which can be explained either by DCs internalizing tumor debris containing TAAs and cross-priming CD8+ T-cells or TLR3 signaling facilitating chemotaxis of the pre-existing CTLs through cytokine production (37, 94).

For many years brain tumors were thought to be resilient to immunotherapy mostly due to the presence of blood-brain barrier (BBB) protecting the brain from harmful substances. However, even though antibodies cannot cross BBB of a healthy person, the barrier often becomes leaky in brain cancer patients (95). However, BBB disruption is usually not uniform, with parts of tumor having intact BBB (96). Additionally, bevacizumab, FDA-approved anti-VEGF antibody normalizing tumor vasculature, was reported to restore low permeability of BBB in glioblastoma patients (97). What's important, metabolically active cells, including immune cells can cross BBB, and produce antibodies or directly target tumor cells inside the brain. The release of HMGB1 as a result of viral-derived ganciclovir tumor cell death activates TLR2 on DCs (immunogenic cell death), resulting in an efficient processing and cross-presentation of tumor antigens leading to tumor regression in a glioblastoma mouse model (98). This gene therapy approach was based on the delivery of Fms-like tyrosine 3 ligand (Flt3L), which is known to enhance dendritic cell infiltration directly to the brain (99).

HMGB1 may also be released from tumor cells dying upon chemo- or radiotherapy therefore stimulating and TLR4 leading to DC maturation (100). The authors showed that TLR4 expression on DCs is necessary for efficient antigen presentation as breast cancer patients with germline *TLR4* loss-of-function allele relapsed faster after chemo- or radiotherapy than those with normal *TLR4* alleles. Chemotherapy has also been followed by TLR9 agonist treatment that stimulated DCs maturation and induced CTL response against tumor antigens that had previously been ignored by the immune system (101). Chemotherapy can contribute to anti-tumor immunity not only by the release of TLR ligands and antigens from dying cells but also by depletion of regulatory T cells (Tregs). One of the mechanisms that tumors developed to evade the immune system is recruitment and expansion of Tregs, CD4+ lymphocytes characterized by expression of CD25 and forkhead box P3 (FOXP3), which are known to suppress immune response. Infiltration of a large number of Tregs into the tumor is often associated with poor prognosis and their removal can evoke and enhance anti-cancer immune response (102). On the other hand, systemic depletion of Tregs may elicit autoimmune diseases and the presence of Tregs is necessary for allograft survival (103). TLR ligands have been shown to block the suppressive effects of Tregs (104, 105). Moreover, an LPS-activated DC vaccine not only inhibits immunosuppressive effects of Tregs but also converts Tregs into interferon-γ-producing Th1-like effectors (106). This result shows high immunostimulatory capacity of DCs matured in the presence of TLR ligand, therefore proposing a more efficient vaccine that could induce immune rejection (107).

Myeloid-derived suppressor cells (MDSCs), alongside Treg cells, play a major role in immunosuppression and are key drivers of tumor immune evasion (108). In a syngeneic mouse model of colon cancer, treatment with combined TLR7/8 and 9 agonists significantly reduced the number of MDSC as well as increased the number of NK and CD8+ T-cells infiltrating the tumor site (43). This combined treatment was efficient not only in small lesions but also in large, established tumors formed by colon cancer or melanoma cells, which were completely eradicated by this TLR agonist combination.

## Summary

Significant advances have been made in tumor biology, including deciphering some specifics of how TLRs play key roles in anti-cancer immunity and tumor rejection. Both exogenous and endogenous ligands are crucial in anti-cancer immunity, but there is a need to develop novel TLR-stimulating therapies. Much research is needed to clearly elucidate the roles of TLR expression in modulating cancer immunotherapy and clinical outcomes. While it is currently unclear, in most cases, which TLR-ligand pairs will produce desired oncological outcomes, there is sufficient evidence to suggest that TLR-modulating therapies will prove to be safe and efficacious for treatment of at least some cancer types. Since cancers are heterogeneous, these new immunotherapies may become integral parts of multi-treatment regimens or may be useful in some cases as monotherapy. Additionally, there is sufficient evidence to suggest that TLR ligands may be useful as immunotherapy adjuvants to increase treatment efficacy and improve patient outcomes. It is difficult to predict whether TLR ligands used as treatments could potentially promote oncogenesis or tumor proliferation; however, the hallmarks of cancer are multi-faceted and often require multiple stimuli to generate tumors. The available data suggest that TLRs play complex functional roles in tumor biology and sometimes act as a double-edged sword in immunotherapy (Figure 1). More studies are needed to unravel roles of TLRs in cancer taking into consideration multiple factors, including TLR (and their types) expression level, mutagenesis, roles of TLR adaptors, and many others. Although tumor heterogeneity is a well-known phenomenon, many researchers do not take it under consideration and study bulk tumors instead of specific populations of cells. Each population of cells (i.e., cancer stem cell, cancer cell, tumor-infiltrating lymphocyte, tumor-associated fibroblast etc.) may have different TLR expression and respond differently to TLR stimulation (25, 26). For example, using melanoma-invaded lymph nodes and melanoma cell lines, Saint-Jean and colleagues showed that melanoma cells expressed TLR 2, 3, 4, 7, and 9 differentially *in vitro* and possibly *ex vivo* (109). While TLR2 and 4 was expressed *ex vivo*, such protein expressions were not observed *in vitro*. In contrast, TLR 3 and 8 proteins were highly expressed *in vitro* but comparatively low expression of these proteins was observed *ex vivo*. Interestingly, TLR 7 and 9 proteins expression was observed in both *ex vivo* and *in vitro* settings. To clearly define, and understand roles of TLRs, it may be better to use isolated cells. However, to translate such mechanistic elucidations into the development of anti-cancer therapeutics, researchers would need to use real-world samples, including bulk tumors (due to tumor heterogeneity). Further studies of TLR's roles and functions in anti-cancer immunity and tumor rejection will greatly advance development of therapeutic interventions to benefit patients undergoing immunotherapy.

## Author Contributions

All authors listed have made a substantial, direct and intellectual contribution to the work, and approved it for publication.

### Conflict of Interest

The authors declare that the research was conducted in the absence of any commercial or financial relationships that could be construed as a potential conflict of interest.

## Abbreviations

TLRs: toll-like receptors
PAMPs: pathogen-associated molecular patterns
DAMPs: danger-associated molecular patterns
TNBC: triple-negative breast cancer
HMGB1: high-mobility group box 1
LPS: lipopolysaccharide
BCG: Bacillus Calmette-Guérin
MPLA: monophosphoryl lipid A
TNFα: tumor necrosis factor alpha
CpG ODNs: CpG oligodeoxynucleotides
CTLs: cytotoxic T lymphocytes
DCs: dendritic cells
HPV: human papillomavirus
Th1: type 1 T helper
NK cells: natural killer cells
NY-ESO-1: New York esophageal squamous cell carcinoma 1
PD-L1: programmed death-ligand 1
TAM: tumor-associated macrophages
MALP-2: macrophage-activating lipopeptide-2
GEMM: genetically engineered mouse model
APCs: antigen-presenting cells
MHC I: major histocompatibility complex I
TCR: T-cell receptor
CD80: cluster of differentiation 80
ROS: reactive oxygen species
MDS: myelodysplastic syndrome
NFκB: nuclear factor kappa-light-chain-enhancer of activated B cells
COX-2: cyclooxygenase-2
MyD88: myeloid differentiation primary response-88
TRIF: TIR-domain-containing adapter-inducing interferon-β
IL: interleukin
IL-1β: interleukin-1 beta
IL-6: interleukin-6
IP-10: interferon gamma-induced protein 10
AP-1: activator protein 1
IRF-3: interferon regulatory factor 3
JNK: c-Jun N-terminal kinase
ERK: extracellular signal-regulated kinase
TIRAP: TIR-domain containing adaptor protein
TRAM: TRIF-related adaptor molecule
DLBCL: diffuse large B-cell lymphoma
STAT3: signal transducer and activator of transcription 3
BTK: burton tyrosine kinase
HDAC: histone deacetylase
M1: classically activated macrophages
M2: alternatively activated macrophages
CXCR3: C-X-C motif chemokine receptor 3
NSCLC: non-small cell lung cancer
TAA: tumor-associated antigen
BBB: blood-brain barrier
Flt3L: fms-like tyrosine 3 ligand
Tregs: regulatory T cells
FOXP3: forkhead box P3
MDSCs: myeloid-derived suppressor cells.
